# Preconception and Prenatal Environmental Factors Associated with Communication Impairments in 9 Year Old Children Using an Exposome-Wide Approach

**DOI:** 10.1371/journal.pone.0118701

**Published:** 2015-03-04

**Authors:** Colin D. Steer, Patrick Bolton, Jean Golding

**Affiliations:** 1 MRC integrative epidemiological unit, School of Social and Community Medicine, University of Bristol, Bristol, United Kingdom; 2 King’s College London, Institute of Psychiatry, London, United Kingdom; 3 Centre for Child and Adolescent Health, School of Social and Community Medicine, University of Bristol, Bristol, United Kingdom; Sun Yat-sen University, CHINA

## Abstract

Although speech and language deficits are common in children and strongly associated with poor educational and social outcomes, little attention has been paid to the antecedents. In this study we used the information from the Avon Longitudinal Study of Parents and Children to examine preconception and prenatal environmental risk factors that were related to communication difficulties in children using the Children’s Communication Checklist (CCC). We used an exposome-wide approach to identify environmental factors univariably associated with the CCC. Taking account of the False Discovery rate, we used a P value of 0.000157 to identify 621 of 3855 items tested. These were then subjected to a series of stepwise linear regression analyses, firstly within 10 domains: personal characteristics, health, development, education, socio-economic variables, lifestyle, home and social environments, life events and chemical and other exposures; and then with the predictive variables from each domain. The final model consisted of 19 variables independently associated with the communication scale. These variables suggested 6 possible mechanisms: stressors primarily associated with socio-economic disadvantage although other lifestyle choices such as a social network of family or friends can ameliorate these effects; indicators of future parenting skills primarily associated with aspects of parental personality; aspects of the home environment; poor maternal health with a novel finding concerning maternal hearing loss; and maternal education which was partially mediated by the child’s IQ. Finally, there may be a mechanism via the maternal diet in pregnancy in particular the consumption of fatty or processed foods. This is the subject of ongoing investigation.

## Introduction

Communication impairments relate not only to the fluency and intelligibility of speech but also to expressive and receptive language impairments. Language impairments can include those involving semantics, such as subtle nuances, as well as pragmatic aspects of language in a social context [1]. Twin studies have suggested that, although children must be exposed to language in their environment in order to learn it, communication impairments have a major heredity component [2,3]. However, the low estimates of environmental influences may be misleading for two reasons. First, these studies may underestimate the importance of environmental effects due to gene-environment interactions being subsumed into the heritability term [4]. Second, different impairments may have varying genetic components. Hence, while speech impairments may have high heritability, language impairments may be largely environmentally determined [5].

A few studies have assessed prenatal exposures that may have an effect on the development of effective communication in the child. Positive associations have been demonstrated for maternal smoking in pregnancy with lower language scores in longitudinal studies of children aged 3–12 years [6–8], as well as in a case-control study of children with specific language impairment [9]. Among studies of illicit drugs, there have been reports of associations between lower general language scores and maternal use of cocaine in pregnancy [10,11]. A study of anticonvulsants used prenatally among women with epilepsy demonstrated an association between phenytoin and both the verbal comprehension and expressive language scores on the Reynell scales, which was not found with a different anticonvulsant [12]. Another study found an association between sodium valproate exposure in the 1^st^ trimester with language impairment [13]. A comparison of 5–6 year olds whose mothers had had prenatal iodine supplementation in an iodine-deficient area in China showed that delay in expressive language was reduced compared with non-supplemented controls [14]. Biomarkers have also shown evidence of possible prenatal mechanisms—for example examination of cord blood of girls in the Raine study showed an association between testosterone levels and subsequent pragmatic language score of the child [15]. However, most of these studies were either based on relatively small numbers or failed to take account of socio-economic and other factors that may have influenced the associations; additionally they did not distinguish specific language difficulties from those associated with more general developmental delay.

In the present study we take advantage of the prospective data collected in the Avon Longitudinal Study of Parents and Children (ALSPAC) which included questions from the Children’s Communication Checklist (CCC), specifically designed to quantify communication difficulties that affect everyday life and which may not be detectable by standardised tests [1]. Here we begin by considering factors prior to the birth of the study child reflecting aspects of the grandparents and parental histories starting from their respective births. These data have the advantage that parental responses are collected before the parents can detect any problem in their child, and hence are not influenced in their responses by any stress their child’s condition may initiate. Other more objective data from biosamples during pregnancy are also analysed.

Since the preconception and prenatal environmental factors that may influence communication impairments are largely uncharted, we use an exposome-wide approach [16]. This is similar to the approach taken in genome wide association studies (GWAS), being hypothesis free. Rather than testing already formulated hypotheses, this approach examines associations to generate hypotheses for replication in other studies. Although largely considered for assays of biological markers [16,17], it has recently been expanded to include lifestyle (such as diet, smoking and exercise) and the dynamic interaction with our surroundings [18,19]. Here we expand the concept further to look additionally at questionnaire based information on the environment in its broadest sense including aspects of parental and grandparental psychosocial traits and health.

## Methods

### ALSPAC sample

The prospective longitudinal cohort study, ALSPAC, was designed with the specific aim of identifying environmental and genetic factors, and the interaction between them, that influence the health and development of the child. Information was collected from around 14000 pregnant women, resident in the county of Avon, with expected date of delivery between 1^st^ April 1991 and 31^st^ December 1992 [20].

### Ethics statement

Ethical approval for the study was obtained from the ALSPAC Ethics and Law Committee and the Southmead, Frenchay, UBHT and Weston Research Ethics Committees. Written consent was obtained from participants to allow use of anonymised linked biological data for research by bona fide scientists.

### Children’s Communication Checklist

**Fig 1:**
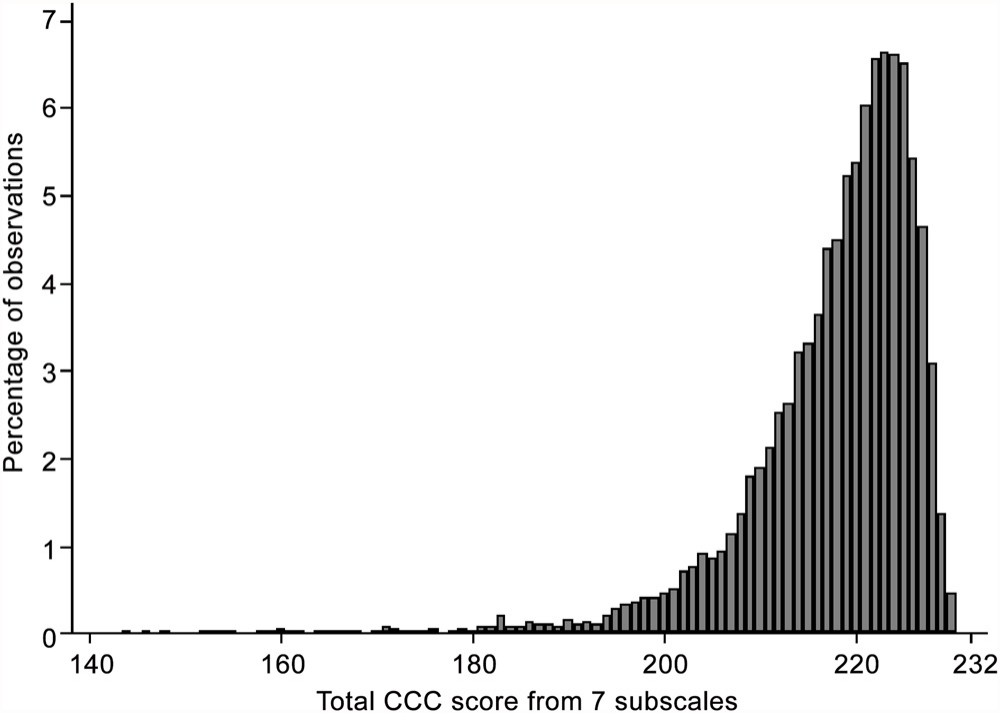
Distribution of the Total CCC Score (N = 7942).

At age 9 the study mother completed a questionnaire which included 7 of the 9 scales of the first version of the Children’s Communication Checklist (CCC) [1]. This checklist was designed to assess aspects of communication that are not readily assessed by conventional standardised tests including aspects of speech and syntax, as well as pragmatic aspects such as over-literal interpretation of stereotyped language. Although the CCC was initially designed to identify pragmatic difficulties, it has been shown to be good at discriminating a wide range of language and communication problems from typical development [21]. Questions were not administered on the subscales *Social relationships* and *Interests*. The present analysis uses a total CCC score obtained by summing the 7 communication scales, with higher scores indicating more typical behaviour. The score had a skewed distribution (Fig. 1). Although the total score had a feasible range of 126–232, no children were observed with the most extreme scores (observed range 144–230).

## ALSPAC exposome

Version 3.01 of the exposome scan includes 3965 variables relating to the preconception and prenatal period as well as details of the study parents and their family background collected from questionnaires completed by the parents (see S1 Methods). Some 110 variables such as illicit drug use, schizophrenia amongst study parents and grandparents and parental birth defects, had only the absence of these features reported within the sample defined by the CCC outcome and have been excluded from subsequent analyses. The main variables discussed in this study are described below classified according to 10 domains.

### (i) Personal characteristics of parents

This domain included variables related to personal characteristics such as personality, attitudes and beliefs.

*Babies need stimulation to develop*—assessed on a 4-point scale ranging from *disagree* to *agree* as reported by the mother.

*Separation anxiety* and *Total sensitivity*—five subscales and a total score derived from the 36 questions on personality using the Interpersonal Sensitivity Measure (IPSM) [22]. High scores indicate greater sensitivity. These two derived scores related to the mother.

*Locus of control score*—a score based upon 12 questions devised to assess whether the individual believes that events resulted primarily from his/her own behaviour (internalised locus) or whether they arose due to chance or fate (externalised locus) [23]. High scores indicate externalising locus. Both maternal and partner reported scores were selected.

*Bottle feeding more convenient*—one of 5 questions concerning attitudes to breastfeeding with a range of responses from *strongly disagree* to *strongly agree*. This question was selected in preference to the combined score.

### (ii) Health of parents

*Night coughing*—absence of, occasional or frequent coughing at night by the mother over the last 2 years.

*Any hearing loss*—derived from any reported problems (*occasional problems (eg infections or glue ear)* to *I cannot hear much at all*) in either ear for the mother.

*Maternal Depression*—measured depressive symptoms using either the 10 items of the Edinburgh Postnatal Depression Scale (EPDS) [24] or the 8 items from the Crown-Crisp (CCEI) scale [25]. At 18w gestation, both derived scores were selected but in addition two individual items from the CCEI were also included. At 32w gestation, only one item from the EPDS (*unnecessary self blame*) was selected.

*Somatic symptoms*—two items from the CCEI at 18w and one item at 32w were selected as reported by the mother.

*Feel good score*—7 questions on subjective health, for instance how irritable or nervous, were combined to create this score. Separate scores relating to the mother as reported by herself and her partner were included. One question relating to the partner (looking attractive as reported by the mother) was also selected. An additional variable also assessed how these 7 traits changed since the mother became pregnant as reported by the partner.

### (iii) Development

This domain is mostly populated by variables relating to the child’s development. However there were a few questions assessing the parents’ own childhood development. *Mother received child guidance* was selected.

### (iv) Education

Variables related to the achievement of national qualifications at 16y and 18y for the mother and a university degree for the partner were selected. *Mother has no qualification* and *Partner has unknown qualification* were also included. All information was reported by the mother. A composite variable of the mother’s highest qualification was also selected but is typically used as an indicator of socio-economic status (see (v) below).

### (v) Socio-economic

*Maternal social class*—ranging from class I (professional /executive) to class V (unskilled) [26]

*Maternal education*—was the highest educational level obtained by late pregnancy. The qualifications obtained were scored on a 5-point scale from the lowest (none or CSE) to highest (University degree).

*Financial difficulties*—a scale derived from maternal reports on the difficulty the family had in affording items such as food, housing, heating, clothes and items for the baby.

*Lowest level of accommodation*—ranged from basement to 16^th^ floor.

*Frequency of car use*—usage by the mother on a 3-point scale ranging from *never* to *everyday*.

### (vi) Lifestyle

There were 11 variables selected. These related to the mother mainly in the 3^rd^ trimester. Of these 7 related to diet.

*Cola intake*—number of cans of cola drunk in 1^st^ trimester by mother

*Drinking of milk*, *total*—the number of glasses drunk on weekdays and weekends at 18 weeks gestation.

*Use of sterilised milk*—with responses *never*, *sometimes* and *usually*.

*Eating offal*—the eating of liver, heart, kidney or liver pate with five responses ranging from *never or rarely* to *more than once per day*.

*Health conscious dietary factor* and *Processed dietary factor*—Maternal dietary patterns were derived from the Food Frequency Questionnaire at 32 weeks gestation using principal component analysis [27]. Of the 5 dietary factors identified from this analysis, two were significant—*health conscious diet* comprising a diet with higher consumption of salad, fruit, rice, pasta, breakfast cereals, fish, eggs, pulses, fruit juices, white meat and non-white bread; and a *processed food diet* which was associated with increased intake of high fat processed foods such as sausages, meat pies and various fried foods.

*Over eating*—responses included *no not at all*, *yes occasionally* and *yes mostly*.

There were also three variables selected concerning the physical activity of the mother.

*Activity comparison*—mothers were asked to rate their physical activity compared to other pregnant women of the same age. Responses ranged from *much less* to *much more*.

*Lift & carry heavy objects*—as reported in the 3^rd^ trimester.

*Usually get in car*—one of four questions concerning main method of transport. Other methods included cycling and public transport with only walking appearing amongst the 621 variables.

In addition there was one variable related to smoking cigarettes in the 3^rd^ trimester by the mother.

### (vii) Home environment

There were five variables selected relating to the mother’s home during childhood.

*Memories of childhood 0–5y*—responses ranged from *very unhappy* to *very happy*.

*Grandmother in household 0–5y*—yes/no.

*Allowed to do as like*—one item from the 10 question overprotective subscale of the Parental Bonding Instrument [28]. The combined subscale score and the other subscale, *maternal care*, were present only in the first selection of 621 variables.

In addition, there were three questions related to the current home environment including aspects of the parental relationship.

*Total number rooms*

*Mother listens to partner*—five responses ranging from *never* to *almost always* as reported by partner.

*Family adversity index*—is a scale combining a variety of adverse factors including indicators of teenage pregnancy/early parenthood, low parental education, housing inadequacy, financial difficulties, large family, poor partner relationships (physical or emotional cruelty or a total lack of support), poor social network (no one with which to discuss problems or help financially), maternal psychopathology (anxiety, depression or attempted suicide) and parental anti-social behaviour (substance abuse or in trouble with the police). Although some of the individual measures comprising this score are discussed elsewhere, this score only used dichotomised indicators of each measure, typically based on the worst 10%. While using binary indicators may potentially lose the predictive power associated with less extreme scenarios, the combined score of these indicators may model the cumulative effect of a number of sources of adversity better than the individual measures.

### (viii) Social environments

These variables relate to the work, neighbourhood and school (when the mother was a child) environments, to discrimination and to social support from friends, relatives and neighbours.

*Bending a lot* and *Mentally demanding tasks*—These two variables were part of a wider range of activity questions which also included whether or not the working day involved a lot of sitting, standing, repetitive tasks or physical exertion. These were assessed pre-, early and mid-pregnancy. The selected variables related to pre-pregnancy.

*Neighbours argue with mother*—assessed using a 5-point scale with responses from *never* to *always*.

*Vandalism worries*—from four questions on parental concerns about the neighbourhood relating to this offence and additionally sex assault, burglary and mugging. Only burglary was absent from the first stage selection of 621 variables.

*School was valuable* and *Often absent from school 11–16y*—the former was assessed on a 5-point scale from *no not at all* to *yes generally* while the latter involved a yes/no response.

*Social network score*—based upon 10 questions asking about the number of friends or relatives with whom the mother had social contact, can discuss problems or can ask for help in times of difficulty during pregnancy. Based upon partner report, the overall combined score failed to meet the domain specific models but an individual item, *number of people to borrow money from*, was included in the 77 variables. Also included was *no one to share feelings* which assessed support as perceived by the mother on a 4-point scale.

*Verbal discrimination*—this was assessed on a 3-point scale relating to the last year as assessed in the 3^rd^ trimester.

### (ix) Life events

These events include becoming pregnant (and variables associated with this), accidents, abuse and variables related to leaving the parental home.

*Maternal age*—age in years at last menstrual period.

*Mother didn’t want this pregnancy*—as reported by the partner in mid-pregnancy.

*Want to know basics about labour*—yes/no, asked at 32w gestation.

*Badly scalded*—derived from a question concerning any occurrence in the past. The responses *Yes stayed in hospital*, *Yes saw GP* and *Yes treated at home* were combined. The other response was *No did not happen*.

*In LA care*—both maternal and partner report of their experience of being in local authority (LA) care.

*Left home <18y* as reported by the mother.

*Mother physically abused 0–16y*—yes/no, retrospective assessment at age 33m of the study child.

*Life events score*—a total of 41 adverse events experienced by the mother or partner during the first half of the pregnancy. In addition to the total number of events as reported by the mother, a number of individual events reported by both parents were also present in the 621 variables including criminal behaviour by the partner and major financial problems.

### (x) Chemical and other exposures

*Total time on the contraceptive pill*—5 responses ranging from *Never used*, *less than one year*, *1–2 years*, *3–4 years* and *>5 years*.

*In smoky room during weekend*—a measure of passive smoke exposure with 5 responses ranging from *never* to *always*.

*Use of photocopier/fax*—one of 12 questions asked during early pregnancy concerning the current use of electrical equipment. Other variables relating to vacuum cleaners, food mixers and PCs were not selected at the 2^nd^ stage.

*Heat water by electricity*—yes usually/no.

*Electrical wiring at work*—one of 19 work related exposures. This and the use of metal cleaner were the only variables part of the 621 variables selected on univariable associations. Other exposures such as dental amalgam, dyes and insecticides failed the FDR criterion in univariable analyses.

*Use carpet cleaner*—one of 16 household chemicals reported by the mother or partner. Although both variables passed the FDR criterion, the partner related variable was selected for domain specific models.

All variables were coded so that high scores reflected more, or the presence, of the particular characteristic. Hence, *Night Coughing* was coded such that the higher score reflected the occurrence of nocturnal coughing; higher scores for *Family adversity index* indicated more adversities.

## Statistical analyses

In primary analyses, linear regression of the CCC score was used with all variables being assumed to be linearly related although these assumptions were explored further in supplementary analyses. Variable selection occurred in three stages. First, variables were assessed by their univariable association with the CCC score after correction for multiple comparisons using the False Discovery Rate (FDR) method [29]. Due to the large sample size associated with the ALSPAC study, it is possible to detect relatively small effects. To identify the more major factors, in terms of variance explained, a stringent family wide error rate of 0.1% was used. It is recognised that the use of statistical significance alone may prejudice the selection of factors with large effects on CCC scores but nevertheless have low explanations due to the rarity of exposure. Subsequent selections reflected multivariable analyses. In stage 2, variables within each domain were considered to form domain specific models. In stage 3, the domain specific models were combined and reduced to form a final model. Multivariable evaluation was made using stepwise techniques. The FDR criterion was used as the criterion for inclusion or removal. This multi-stage approach was considered preferable to a global stepwise model using all stage 1 factors since variables in the final model should also be important predictors in a domain specific model. Missing values were imputed using the method of chained equations [30]. Imputations were performed using all stage 1 selected variables for 13,971 children surviving to age 5 years. It was considered preferable to utilise as much data as possible to improve the precision of the imputed estimates although the number available with observed CCC data (used in subsequent analyses) would only be 7613 children. Variables were standardised to have a variance of 1 to facilitate comparison of effect sizes. The ranking of variables in unadjusted associations was by the variance explained.

Partner variables were given special consideration in the analyses. For the 2.0% of mothers without a partner, these variables were set to an arbitrary value [31]. To differentiate between these assumed values and observed data (with the same value), it was important to include a partner status variable in the model. The effect of no current partner on the CCC score will vary according to the assumed value and how similar no partner is to a current partner with the same response. In this study the assumed value reflected the lowest feasible value (zero). Further details about the treatment of partner variables are given in S2 Methods.

To explore the final model more fully, additional analyses were performed. First, interactions between pairs of exposure variables were assessed. These derived variables were generated as the product of exposure variables in a particular pair. Interactions were adjusted for all factors in the final model. Second, non-linear associations were investigated by including quadratic terms for each variable. A final analysis was conducted to explore whether risk factors were specific for communicative ability, or associated more generally with intellectual developments as measured by WISC performance and verbal IQ assessed for the child at 8 years [32]. Similar mediation analyses were conducted for birth weight. These analyses were performed by entering the mediators into the final model and exploring the impact on their effect sizes.

The robustness of the final model was explored in sensitivity analyses. These analyses explored sensitivity to violations of the normality assumption by the use of ordinal regression, to the model selection procedure, to the imputation of data and to the use of linear regression by comparison with decision tree analysis (CHAID) [33]. Decision tree analysis attempts to classify the children’s CCC scores into homogenous groups as defined by predictor variables. It is analogous to regression analyses where the predictors have non-linear associations forming branches and where interactions exist between predictors forming the hierarchical tree. The exploration of model consistency was evaluated by comparisons of the results from forwards, backwards and subset stepwise techniques and changes to the inclusion/removal criteria (see S3 Methods). In addition, results from the use of orthogonal derived variables from a factor analysis were compared to those from the original 621 correlated variables.

While this paper was hypothesis free, we include an example of how this methodology could be extended to a scenario where some prior hypotheses existed. To illustrate this extension to the standard analyses, parental locus of control was selected.

## Results

### Multiple comparisons

**Fig 2:**
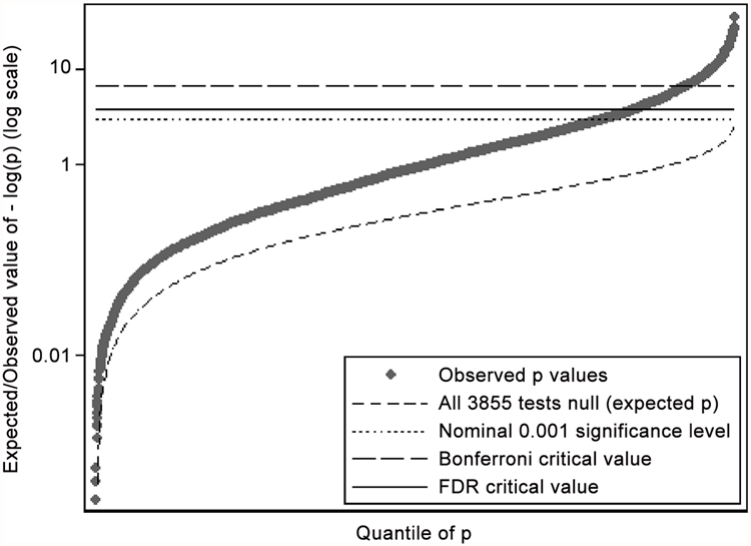
Q-Q plot of the p values from the analysis of all 3855 variables. The False Discovery Rate (FDR) method for multiple comparisons utilises a variable criterion ranging from the nominal significance level for the highest p value to the equivalent Bonferroni critical value for the lowest p value. The specific FDR criterion for a particular set of results is determined where this line intersects the observed p values. For these results, the FDR critical value was 0.0001572.

The FDR method suggested a nominal p value of 0.0001572 as the criterion for significance (see Fig. 2). In total, 621 variables had univariable associations which passed this criterion. Domains differed in the proportion of variables meeting the FDR criterion ranging from 38% for the socio-economic environment to 5% for life events (see Table 1).

**Table 1:** Selection of variables in the analysis of the total CCC score.

		Number variables		Domain models		Final
Domain		Total	Selected	df	R^2^	Model
I	Personal characteristics	353	88 (24.9%)	11	9.62%	6
II	Health	828	145 (17.5%)	13	7.85%	4
III	Development	14	2 (14.3%)	1	0.44%	0
IV	Education	120	15 (12.5%)	5	4.14%	0
V	Socio-economic	80	30 (37.5%)	6	6.83%	2
VI	Lifestyle	431	90 (20.9%)	11	6.68%	1
VII	Home environment	440	73 (16.6%)	6	6.95%	0
VIII	Social environment	242	80 (33.1%)	10	8.23%	3
IX	Life events	836	43 (5.1%)	8	4.41%	3
X	Chemical and other exposures	511	55 (10.8%)	6	4.54%	0
	Total	3855	621 (16.1%)	77		19

Variables were initially selected based upon univariable associations (p<0.00016). This criterion was determined by the False Discovery Rate method. These 621 variables were further selected based upon domain specific stepwise models (see Table 2). The variance explained by these models ranged from <1% to <10%. The 77 variables from the 10 domains were then reduced using stepwise regression to produce the final model (see Table 3). The R^2^ of this model was 13.34% and included variables from six domains.

**Table 2:** Unadjusted and adjusted associations for the 77 variables present in domain specific models.

Domain	Variable	Unadjusted	Adjusted within domain
		B [95% CI]	B [95% CI]
I	Separation anxiety	-1.53 [-1.73,-1.32]	-1.34 [-1.71,-0.97]
	Bottle feeding more convenient	-1.24 [-1.45,-1.04]	-0.61 [-0.82,-0.41]
	Babies need stimulation to develop	1.02 [0.81,1.23]	0.58 [0.37,0.78]
	Others will think less of real self^a^	-1.20 [-1.41,-0.99]	-0.63 [-0.87,-0.39]
	Partner Others wouldn’t like true self^a^	-0.71 [-0.92,-0.50]	-0.39 [-0.59,-0.19]
	Total sensitivity	-0.62 [-0.83,-0.41]	0.92 [0.57,1.27]
	Often unfairly blamed^b^	-1.74 [-1.94,-1.53]	-0.75 [-0.97,-0.52]
	Effort would be in vain^b^	-1.74 [-1.94,-1.53]	-0.67 [-0.89,-0.44]
	School effort useless^b^	-1.30 [-1.50,-1.09]	-0.54 [-0.75,-0.33]
	Partner Locus of control score	-1.27 [-1.48,-1.06]	-0.67 [-0.88,-0.45]
	Want to know basics about labour	-0.96 [-1.17,-0.75]	-0.47 [-0.68,-0.27]
II	Night coughing in past 2y	-1.10 [-1.31,-0.89]	-0.65 [-0.85,-0.44]
	Unnecessary self blame^c^	-1.50 [-1.71,-1.30]	-0.69 [-0.92,-0.46]
	EPDS depression 18w	-1.77 [-1.98,-1.57]	-0.75 [-1.05,-0.45]
	Need to cry^d^	-0.73 [-0.94,-0.52]	0.75 [0.47,1.04]
	Need special effort for crises^d^	-0.59 [-0.80,-0.38]	0.61 [0.35,0.86]
	CCEI Depression 18w	-1.47 [-1.68,-1.27]	-0.75 [-1.10,-0.40]
	Falling asleep OK 12w^e^	1.21 [1.00,1.41]	0.47 [0.26,0.69]
	Loss of appetite 12w^e^	-1.06 [-1.27,-0.86]	-0.43 [-0.64,-0.22]
	Tingling or prickling sensations 32w^e^	1.09 [0.88,1.30]	0.45 [0.24,0.66]
	Feel good score	1.63 [1.42,1.84]	0.69 [0.44,0.93]
	Partner change in Feel Good score	-0.72 [-0.97,-0.46]	-0.70 [-0.95,-0.45]
	Partner looking attractive	1.01 [0.80,1.22]	0.48 [0.27,0.69]
	Any hearing loss	-0.67 [-0.88,-0.46]	-0.41 [-0.61,-0.21]
III	Child guidance in childhood	-0.62 [-0.83,-0.41]	-0.62 [-0.83,-0.41]
IV	Has O level	1.51 [1.30,1.72]	1.00 [0.77,1.23]
	Has no qualification	-0.97 [-1.18,-0.76]	-0.53 [-0.74,-0.31]
	Partner has unknown qualification	-0.76 [-0.96,-0.55]	-0.49 [-0.70,-0.28]
	Partner has university degree	1.03 [0.82,1.24]	0.52 [0.29,0.75]
	Has A level	1.24 [1.03,1.44]	0.54 [0.30,0.79]
V	Maternal education	1.81 [1.60,2.01]	1.11 [0.86,1.35]
	Difficulty in affording things for baby	-1.42 [-1.62,-1.21]	-0.96 [-1.17,-0.75]
	Lowest level of accommodation	-1.03 [-1.23,-0.82]	-0.66 [-0.86,-0.45]
	Frequency of car use	1.31 [1.11,1.52]	0.59 [0.38,0.81]
	Maternal social class	-1.57 [-1.78,-1.37]	-0.52 [-0.76,-0.27]
	Major group of last job	0.79 [0.58,1.00]	0.41 [0.20,0.61]
VI	Health conscious factor score	1.42 [1.22,1.63]	1.00 [0.79,1.22]
	Processed factor score	-1.06 [-1.27,-0.85]	-0.67 [-0.88,-0.46]
	Loss of control over eating	-0.83 [-1.04,-0.62]	-0.63 [-0.83,-0.42]
	Activity comparison	0.83 [0.62,1.03]	0.59 [0.38,0.79]
	Usually get in car	0.83 [0.62,1.04]	0.53 [0.33,0.74]
	Lift & carry heavy objects	-0.63 [-0.84,-0.42]	-0.51 [-0.71,-0.30]
	Smoked cigarettes 3^rd^ trimester	-1.12 [-1.33,-0.91]	-0.52 [-0.74,-0.31]
	Total milk intake	-0.73 [-0.93,-0.52]	-0.48 [-0.69,-0.28]
	Cola intake	-1.04 [-1.25,-0.83]	-0.50 [-0.71,-0.29]
	Frequency of eating offal	-0.57 [-0.78,-0.36]	-0.41 [-0.62,-0.21]
	Use sterilised milk	-0.53 [-0.74,-0.32]	-0.40 [-0.60,-0.20]
VII	Family adversity index	-1.99 [-2.19,-1.78]	-1.46 [-1.68,-1.24]
	Grandmother in household 0–5y	0.98 [0.77,1.19]	0.62 [0.42,0.83]
	Mother listens to partner’s feelings	1.19 [0.98,1.40]	0.61 [0.40,0.82]
	Total rooms	0.95 [0.74,1.16]	0.58 [0.38,0.79]
	Allowed to do as liked 0–16y	1.10 [0.89,1.30]	0.58 [0.37,0.79]
	Memories of childhood 0–5y	1.14 [0.93,1.35]	0.54 [0.32,0.75]
VIII	Social network score	1.68 [1.47,1.88]	0.87 [0.64,1.09]
	No one to share feelings	-1.52 [-1.72,-1.31]	-0.74 [-0.96,-0.52]
	School was valuable experience	1.35 [1.15,1.56]	0.68 [0.46,0.90]
	People to borrow money from	1.36 [1.16,1.57]	0.65 [0.44,0.86]
	Mother argues with neighbours	-0.91 [-1.11,-0.70]	-0.53 [-0.74,-0.33]
	Bending a lot pre-pregnancy	-0.75 [-0.95,-0.54]	-0.52 [-0.72,-0.32]
	Demanding tasks pre-pregnancy	1.00 [0.79,1.21]	0.50 [0.29,0.70]
	Verbal discrimination	-0.88 [-1.09,-0.68]	-0.48 [-0.69,-0.28]
	Often absent from school 11–16y	-1.23 [-1.44,-1.02]	-0.49 [-0.71,-0.27]
	Vandalism worries	-0.86 [-1.06,-0.65]	-0.45 [-0.65,-0.25]
IX	Maternal age last menstrual period	1.01 [0.80,1.21]	0.87 [0.66,1.07]
	Physically abused 0–16y	-1.02 [-1.23,-0.81]	-0.67 [-0.88,-0.46]
	Life events score	-0.98 [-1.19,-0.77]	-0.63 [-0.85,-0.42]
	Mother didn’t want this pregnancy	-0.75 [-0.96,-0.54]	-0.53 [-0.73,-0.32]
	Ever badly scalded	-0.62 [-0.83,-0.41]	-0.49 [-0.70,-0.29]
	In local authority care	-0.77 [-0.98,-0.56]	-0.48 [-0.69,-0.27]
	Partner in local authority care	-0.66 [-0.87,-0.45]	-0.42 [-0.63,-0.21]
	Left home before 18yrs old	-0.76 [-0.97,-0.55]	-0.41 [-0.62,-0.20]
X	In smoky room during weekday	-1.35 [-1.56,-1.14]	-1.10 [-1.30,-0.89]
	Photocopier or fax use	-1.07 [-1.28,-0.87]	-0.92 [-1.13,-0.72]
	Partner carpet cleaner use pre-preg	-0.79 [-1.00,-0.58]	-0.65 [-0.85,-0.44]
	Usually heat water by electricity	-0.81 [-1.02,-0.60]	-0.55 [-0.76,-0.34]
	Electrical wiring	0.55 [0.34,0.76]	0.47 [0.26,0.67]
	Total time on the contraceptive pill	0.61 [0.40,0.82]	0.42 [0.21,0.63]

Variables ranked by partial correlation within each domain. All variables relate to the mother unless specified otherwise. In unadjusted analyses, all p values were <0.00000085; in adjusted analyses p<0.000153. Variables have been standardised to have a variance of 1. The Partner status variable was important for domains I, IV and X.

^a^ Part of Fragile inner self subscale at 18w

^b^ Part of maternal locus of control at 12w

^c^ Part of maternal EPDS at 32w

^d^ Part of maternal CCEI depression subscale at 12w

^e^ Part of maternal CCEI somatic subscale

**Table 3:** Final model of 19 variables derived from the 77 independently associated variables from domain specific models by linear and ordinal regression (N = 7613).

Variable	Linear	Ordinal logistic
	B [95% CI]	OR [95% CI]
Maternal education	0.79 [0.57,1.01]	1.25 [1.19,1.30]
Social network score	0.70 [0.49,0.90]	1.16 [1.11,1.20]
Feel good score	0.60 [0.39,0.81]	1.15 [1.10,1.20]
Others will think less of real self ^a^	-0.56 [-0.77,-0.35]	0.92 [0.88,0.96]
Unnecessary self blame ^b^	-0.56 [-0.77,-0.35]	0.85 [0.82,0.89]
Lowest level of accommodation	-0.51 [-0.71,-0.31]	0.94 [0.90,0.97] ^f^
Often unfairly blamed ^c^	-0.51 [-0.72,-0.29]	0.88 [0.85,0.92]
Babies need stimulation to develop	0.48 [0.28,0.68]	1.12 [1.08,1.17]
Physically abused 0–16y	-0.45 [-0.65,-0.25]	0.93 [0.89,0.97] ^f^
Processed dietary factor	-0.45 [-0.65,-0.25]	0.91 [0.88,0.95]
Mother argues with neighbours	-0.44 [-0.64,-0.24]	0.91 [0.87,0.94]
Bending a lot pre-pregnancy	-0.43 [-0.63,-0.24]	0.92 [0.88,0.95]
Effort would be in vain ^c^	-0.47 [-0.68,-0.25]	0.92 [0.88,0.96] ^f^
Mother didn’t want this pregnancy ^d^	-0.41 [-0.61,-0.22]	0.94 [0.90,0.98] ^f^
Bottle feeding more convenient	-0.43 [-0.64,-0.23]	0.91 [0.87,0.95]
Want to know about labour	-0.40 [-0.60,-0.20]	0.93 [0.89,0.96] ^f^
Night coughing in past 2y	-0.40 [-0.60,-0.20]	0.92 [0.88,0.96]
Ever badly scalded	-0.38 [-0.58,-0.18]	0.93 [0.90,0.97] ^f^
Any hearing loss	-0.39 [-0.58,-0.19]	0.93 [0.90,0.97] ^f^

Variables are ranked by their partial correlation in linear regression. This is almost equivalent to ranking by effect size. Variables have been standardised to have a variance of 1. All p values in ordinal regression <0.0036 with 12 variables p< FDR criterion. The explanations of the log likelihood by the linear and ordinal models were 13.34% and 2.39% respectively. B>0 and ORs >1 are beneficial effects.

^a^ Part of Fragile inner self subscale at 18w

^b^ Part of EPDS at 32w

^c^ Part of maternal locus of control at 12w

^d^ As reported by the partner at 18w

^f^ p value > FDR criterion

### Missing value imputation

Missing data accounted for 16% of the data points in the imputed data set (N = 13971 for 621 variables) and 9% of the data points for children with valid CCC data (N = 7613). Further details are given in S1 Results and S1 Table.

### Domain specific models

In order to eliminate those variables whose association with CCC was confounded with others, backwards stepwise linear regression was applied to each domain (see S2 Results and S2 Table). This process identified 77 variables which have been described in the Methods section and summarised in Table 1.

The unadjusted associations of the 77 variables are shown in Table 2. As expected the significance of these associations was much lower than the FDR criterion reflecting that those variables with the strongest univariable associations tended to be important in domain specific models. However the weakest of the associations in Table 2, *Partner’s use of carpet cleaner*, was ranked 583 in all 621 variables indicating that some variables with stronger univariable associations had been eliminated due to their high correlation with other variables in the same domain. The strongest unadjusted association was for the *Family adversity index* (p = 7.0x10^-81^). For five subscales of the IPSM, EPDS and CCEI, individual questions were selected in preference to the composite score (see footnotes to Table 2).

The 10 domain specific models are also shown in Table 2. The average attenuation of the effect sizes for 73 variables due to adjustment was 41% (range 2% to 67%). *Total sensitivity* and two items from CCEI depression subscale increased their effect sizes by 57%, 3% and 3% respectively but also changed sign. [*Mother received child guidance* was the only selected variable in domain III.] There was some tendency for the larger numbers of variables to be associated with the larger attenuations in these domain models. Hence, domains IV, V, VII, IX and X which had the smallest number of variables in their specific models, had the lowest attenuations on average at 36%. Domains I, II, VI and VIII with the largest number of variables had the greatest attenuation of 44% on average. The strongest association in these multivariable models was *Family adversity index* while the weakest was *Others would not like true self*. The 10 domain models explained between 0.4% and 9.6% of the variability in CCC score (see Table 1).

### Final model

Of the 77 variables identified from stepwise regressions within domains, 19 variables were retained in the final model with an R^2^ value of 13% (see Table 3). This model was only identified using subset stepwise regression (see S2 Results and S3 Table). The ranks of these variables, in terms of their unadjusted associations, were in the range 9 to 518. The top five variables (ranks 9 to 23) explained 9.3% (69% of the total explanation for the final model). However a significant contribution was made by the other 14 variables despite their unadjusted associations being relatively minor compared to the top 5 (5 variables with ranks 40 to 93 adding an additional 1.9% (14% of total) and 9 variables with ranks 153 to 518 adding the remaining 2.2% (17%of total)). It is clear that unadjusted associations may not be a good indicator of independent predictive power amongst a set of correlated variables. The *Family adversity index* failed to enter the model despite having the strongest unadjusted association. This may reflect its composite nature including elements of education, maternal depression and social network. Even within the final model, most variables were highly correlated. In total, 33 of the 171 pairwise correlations had p>0.05 but all other correlations were significant with 103 having p<0.0001. In particular, the two items of the locus of control scale (*Often unfairly blamed* and *Effort would be in vain*) had a correlation r = 0.34 (S4 Table).

### Interactions between variables in the final model

**Fig 3:**
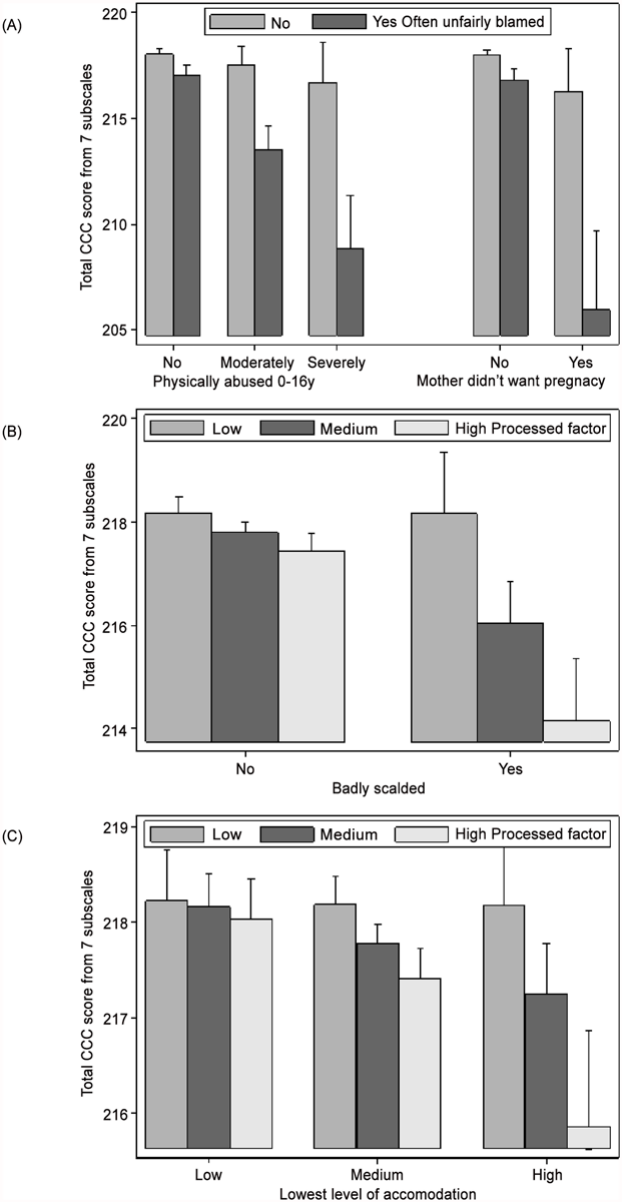
Interaction effects on the Total CCC score (N = 7613). The figure shows four interactions: two with *Often unfairly blamed* (A) and two with *Processed dietary factor* (B and C). The significance of the interactions were p = 0.000019 and 0.000022 (A), p = 0.000071 (B) and p = 0.000048 (C).

Of the 171 pairs of variables tested for interaction, 13 achieved p<0.001 after adjustment for other factors in the final linear regression model. Seven of these related to *Lowest level of accommodation* with three related to *Physically abused 0–16y*, *Social network* and *Processed dietary factor*. Further exploration suggested four interactions could explain all other interactions. These involved *Often unfairly blamed* and *Physically abused 0–16y* (p = 0.000019), *Often unfairly blamed* and *Mother didn’t want pregnancy* (p = 0.000022), *Badly scalded* and *Processed dietary factor* (p = 0.000071), and *Lowest level of accommodation* and *Processed dietary factor* (p = 0.000048) (Fig. 3). These four interactions all reflected a similar pattern of little or no effect for one variable when the other was at the more favourable extreme but a marked effect when at the unfavourable extreme.

### Non-linear effects

In all, 12 of the 19 variables could be tested for non-linear effects. (It was not possible to test the binary *Want to know about labour*, *Often unfairly blamed*, *Effort would be in vain*, *Any hearing loss*, *Mother didn’t want this pregnancy*, *Badly scalded* and *Bending a lot pre-pregnancy* variables). There was no evidence of non-linearity (p>0.001).

### Potential mediators

Adjustment for child performance IQ alone and together with verbal IQ generally had a major impact on the final model. Four particular examples were observed: for *Maternal education*, *Processed dietary factor*, *Bottle feeding more convenient* and *Mother didn’t want this pregnancy*, their effects attenuated by 66%, 39%, 28% and 22% respectively—these results being consistent with no effect (p>0.0023. But for most other variables, their effects were not totally mediated by IQ. Nevertheless, up to 29% of the total explanation may have been mediated via the effect of IQ.

Mediation by birth weight as a dimensional variable appeared to be minimal with effect sizes attenuating by <3.3%. About 1% of the total explanation was mediated by birth weight. This result reflected that, although there were strong associations between birth weight and both the CCC score and the predictors in the final model (p<1.6x10^-6^), the variance explained in both analyses was low (R^2^<0.8%).

### Sensitivity analyses

To assess the sensitivity of the final model to normality assumptions, we repeated the analyses using ordinal regression. This showed similar results to those obtained from linear regression (Table 3). The ranking of the unstandardised effect sizes were similar with no variable changing by more than 3 positions. The statistical significance of effect sizes was generally lower in the ordinal regression analysis. This was most severe for the *Lowest level of accommodation*.

A factor analysis of the 621 variables generated 82 factors. An analysis of the relationship between these factors and the CCC score generally supported the final model using the individual variables. In all, 29 factors were related to the CCC score of which 13 mapped onto 19 variables in the final model. The extra factors suggested other possible effects for parental smoking, relationship with the partner, partner’s health, aspects of the neighbourhood not related to arguments with neighbours, life events in particular financial problems and the stability of the mother’s childhood. Six other factors were related to the six remaining individual variables but these factors only entered the model at a less stringent level than the FDR criterion. Further details are given in S3 Results and S5 Table.

Stepwise models showed greater consistency for imputed compared to observed data sets and for subset regressions (see S2 Results and S2 and S3 Tables). The three variables reported in Table 3 which were not identified by other stepwise methods were *Want to know about labour*, *Night coughing in past 2y* and *Ever badly scalded*. These were three of the four variables with the weakest associations. The imputed data also supported a wider range of predictors compared to models derived from observed data only. This may suggest that any extra random variability introduced by the imputation process was outweighed by the increased sample size. Subset regressions tended to identify superior models in terms of R^2^ than either forwards or backwards techniques.

CHAID analyses supported the role of maternal education, the processed dietary factor and aspects of housing, social network, locus of control and self-esteem (*Fragile inner self* and body image). Despite this general agreement, often the precise variable in these analyses and subset regression differed. In addition, CHAID analyses suggested maternal absence from school and the type of the mother’s last job may also be related to the CCC scores (see S6 Table).

### Prior hypotheses

The exposome scan can be easily modified to include prior hypotheses. The fundamental difference for variables with an expected effect compared to the other variables in the scan is that the same criterion should not be applied. In other words, evidence from other studies can weaken the criteria for inclusion for these particular variables. To illustrate this approach, parental locus of control was selected based upon some evidence of its role in the development of communication skills in children [34,35]. With this adjustment, 490 variables passed the first stage selection. The second stage selection based upon domain specific models identified 63 variables of which 52 with *(Partner) Locus of control* were part of the previous 77 selected at this stage. The new candidates for inclusion in the final model included maternal smoking in the last trimester, vitamin C intake and a household chemical score. The final model was almost identical to the unconstrained model with the exception that the *Eating of offal* replaced the *Processed dietary factor* and *Ever badly scalded*. These analyses supported the role of parental locus of control (p = 6.3x10^-10^ and 0.0045 for maternal and partner variables respectively). It should be noted that the maternal effect was evident through two individual items rather than via the composite score of 12 items (p = 0.855).

## Discussion

To our knowledge this is the first large prospective population study to assess comprehensively the factors identifiable prenatally that are associated with the child’s ability to communicate 9 years later. We used a total CCC score and while this measure includes aspects of phonology, grammar and semantics, it is primarily a measure of pragmatic language [21]. It is to be noted that each of these domains may have distinct environmental associations compared to the total score. We found that, although there were as many as 621 univariable associations with p<0.000157, and many more at less stringent levels of significance, multivariable analyses revealed that most of these were not significant once key facets were taken into account. The 19 variables retained in the final model could be categorised by 6 different criteria which may suggest possible mechanisms:

### (1) Prenatal stress and stress-coping mechanisms

Various studies have demonstrated adverse effects on the offspring of maternal prenatal anxiety and stress using both animal and human studies. The effects include changes in behaviour in utero, as well as in childhood [36,37]. Further evidence of the effects on brain function comes from a study of brain scans. This showed that those 6–9 year old children whose mothers had been very anxious at 19 weeks gestation had reduced grey matter density in various parts of the brain [38]. In spite of the various studies of the effects of maternal stress and anxiety on brain development and birth outcomes, only three studies appear to have examined whether there are effects on language ability. In one study, the authors showed that prenatal stress resulting from power failures due to the Quebec ice storm was negatively associated with language abilities accounting for 17% and 18% of the variation in receptive language at age 2 years and 5½ years respectively [39,40]. The higher R^2^s in these reports compared to this study may reflect their small sample size (N = 58 and 89), the earlier age of the assessment or outcomes which reflected understanding vocabulary rather than the more diverse components within the CCC. Another study investigated children born after prenatal exposure to ionising radiation as a consequence of the Chernobyl accident. The authors found increased levels of impairments in communication in the exposed group but felt that this was more likely to be due to the stress of the event and the subsequent evacuation and relocation rather than the radiation per se [41]. In contrast to these studies on the effect of disasters, a study on more typical stressful events such as financial or relationship difficulties reported no association with receptive language abilities [42].

In our study, we found some supporting evidence. Maternal depression may have served as a proxy for stress [43]. Similarly, arguments with neighbours and an unwanted pregnancy may add to the mother’s stress. In addition, the positive association of the mother’s social network with the CCC score may reflect its ameliorating effect on the negative effects of stress [44].

Prenatal stress has been associated with low birth weight [44] and low birth weight has been associated with poor language attainment [45]. This raised the possibility that the identified prenatal factors may only influence the CCC score via their effect on birth weight. Mediation analyses suggested there was a significant and major direct effect of the prenatal factors with mediation only playing a minor role.

### (2) Maternal health

Nocturnal coughing is a common symptom of asthma with its prevalence in the ALSPAC sample being 30% [46]. While part of the prevalence may be attributable to undiagnosed asthma [47], it seems likely that a large proportion represents sub-clinical respiratory problems. Asthma and depression have been associated with adverse pregnancy outcomes including preterm and caesarean delivery [48–50] which may lead to adverse child development including language [51–53]. A novel finding was the negative association of maternal hearing loss with the CCC score. As many as 14% of the study population reported that they had less than perfect hearing. The mechanism by which maternal hearing loss affects child communication impairments requires investigation but may reflect a genetic link whereby the child has inherited the mother’s hearing loss or alternatively a hindrance to appropriate responses to the child. Our study also suggested that more subjective measures of prenatal health, the *Feel good score*, may also be important.

### (3) Indicators of future parenting skills

The identified maternal personality factors, *Fragile inner-self* and *Locus of control*, are possible markers of parental competence [54,55] with maternal external locus of control being associated with less stimulating home environments [56–58] and lower levels of child preventive health care [59–61]. These studies support our findings of negative relationships between these factors and communication impairments. Furthermore, the mother’s positive attitude in pregnancy to the baby’s future development would be expected to have a positive influence on parenting and subsequently to the CCC score. Similarly, positive attitudes to breastfeeding are likely to favour this method of feeding after the birth with its associated benefits on language development [62]. Less than perfect maternal hearing may also hinder effective parenting. It may be more difficult for the mother to understand their child’s early attempts at speech. Furthermore, their own speech may be less clear and so harder for the child to understand. It should be pointed out that most of these women were not profoundly deaf. Indeed the relationship with the child’s CCC score was numerically greater if the loss was unilateral compared to bilateral, -1.67 [-2.76, -0.57] compared to -1.14 [-1.87, -0.41], although the difference was statistically non-significant (p = 0.42).

A review of the literature has suggested that other factors identified in this study have been associated with parenting: maternal education [63], depression [55,64], social networks [65] and childhood abuse [66]. The evidence of whether parenting affects communication impairments is mixed with some studies showing an association [67,68] but not others [69]. However in the latter study adjustment was made for other neurobiological factors such as fine motor and personal-social skills which may have a common aetiology with late language emergence and hence underestimate the effect of parenting.

### (4) Home environment

While much more detailed information on the home environment was collected after birth, it was interesting to note some variables included in the model which may reflect facets of the home environment. *Lowest level of accommodation* may indicate the availability to play outside in a safe monitored environment such as a garden. *Mother argues with neighbours* may reflect tensions outside the home which nevertheless, if they persist after birth, would have repercussions inside the home. *Bending a lot pre-pregnancy* was prima facie a less easy variable to interpret with its negative effect on the CCC score. However factor analysis showed this variable loaded on the same factor but with an opposite sign to the use of VDUs/PCs. This may suggest a more plausible explanation that a ‘computer literate’ mother would have positive benefits for communication skills.

### (5) Noxious exposures

There is considerable evidence to suggest that chemical and other insults to the fetus during pregnancy can result in damage to the developing brain, demonstrated in particular by developmental changes during infancy. In regard to language development, one of the most striking results in the literature concerns a relationship with maternal prenatal cigarette smoking. Statistically significant results have been shown in the literature with various measures of speech and language development [6,7,9]. In this study we identified 42 measures of parental smoking and smoking by the maternal grandmother during her pregnancy, which passed the FDR criterion univariably, but only one, maternal smoking in the 3^rd^ trimester, entered the domain specific model. Many of the other markers that we used to identify other chemical constituents that might have an adverse effect on the developing brain failed to show independent associations.

The only noxious exposure present in the final model was the *Processed dietary factor* reflecting the consumption of high fat foods. While high fat intakes in pregnancy have been associated with maternal depression [70], its independent association with the CCC score after adjusting for depression suggests a different and as yet unidentified causal pathway in the aetiology of communication impairments. Early results from the ALSPAC study indicate that the mothers eating this type of diet have a different pattern of toxins in their blood (Golding, personal communication). Other mechanisms may involve epigenetics [71] or excessive gestational weight gain leading to intra-uterine stress [72]. Further evidence on the effect of environmental exposures came from supplementary factor analyses. These supported the negative role of smoking.

### (6) Maternal education

Measures of parental education have frequently been considered in relation to language development with most reporting positive associations [6,7,9,10,42] but not [69]. To our knowledge, however, no study appears to have assessed whether this reflected a specific effect on language, independent of a more general effect on verbal and nonverbal cognitive development as shown in this study.

This discussion has described how factors may exert an influence in multiple ways. This may reflect a complex situation whereby more distal factors, such as maternal education, can exert direct influences but also other effects mediated via more proximal factors [73]. Our analyses have also suggested that associations may not act independently but may be moderated by other variables as in the case of maternal locus of control and *Mother didn’t want this pregnancy*. We have also hypothesised how some of the variables may reflect future parenting skills and the future home environment. Further studies with this cohort will assess whether these explanations are valid as their offspring develop. But from an intervention point of view, it is more important to identify the factors as early as possible and to determine their reliability in identifying at risk individuals than whether the precise timing of the effect.

In this study we considered effects across the whole range of the CCC score. In other words we were estimating mean CCC scores for particular groups of children defined by the predictors in Table 3. Since there is random scatter about these means, it is interesting to evaluate how this might affect the prevalence of an impaired group defined as the worse 10% for the whole population. An increase of 3 CCC points (for instance, as predicted from our final model, by improving maternal education from low to high) would change the prevalence to ~7% while a 3 point decrease would increase the prevalence to ~15%.

It is appropriate to question whether the results of these analyses merely reflect the effects of the child’s IQ, since language impairments are known to be more prevalent the lower the IQ. We therefore added the child’s performance IQ to the final model of Table 3. All of the factors retained independent associations at the 5% significance level suggesting that performance IQ may only partially mediate their effects. Adding verbal IQ to this model further attenuated the effect sizes especially for *Processed dietary factor*. Thus, despite substantial mediating effects of IQ, most variables in the final model had residual direct effects on the CCC score. While beyond the scope of this current study, it seems plausible that this mediating effect may suggest the existence of other important environmental exposures in the postnatal period.

We also explored in this study how an exposome scan can be modified to incorporate prior hypotheses such as parental locus of control. In this particular example, the final model was very similar to the unconstrained model. However, in general, this cannot be assumed and may lead to more substantial differences in final models using other prior hypotheses.

It is also interesting to note that in some cases individual items of composite scores were selected in preference to the composite score such as occurred for maternal locus of control. Whether this is a chance event or a reflection that the score is not measuring a unidimensional construct is unclear. But results from a factor analysis may suggest the latter.

While the exposome-wide approach has many similarities to GWAS, it is important to recognise key differences. Unlike GWAS, where individuals are exposed to their genes throughout life, the exposome will include a variety of exposures assessed at different times including repeat measures of the same exposure. This raises issues concerning the modelling of the cumulative effect of these exposures with possible nonlinear effects due to chronic compared to acute exposures or critical time periods. As a starting point, this study has concentrated on the preconception and prenatal period. It also has to be noted that there are usually multiple strong correlations between the environmental exposures, particularly those related to socioeconomic factors. This contrasts with GWAS where the genetic variants are largely independent when related to different genes although within genes there is often strong linkage disequilibrium. Consequently any factors identified in an exposome scan must be analysed multivariably on the assumption that the true causal factors are likely to have stronger associations than those which are co-incidentally associated. This was achieved by stepwise regression in this study. Replication of results is important to both methods but there are distinct difficulties with the exposome approach. Whereas any cohort can obtain GWAS data by collecting DNA from its participants even if such analyses were not envisaged at the outset, it is not so easy to collect missing exposure data at earlier times without introducing recall bias from retrospective collection. In addition, whereas genetic variants have a standard set of ‘responses’ (the genotypes), subtle differences in environmental assessments can be introduced by the wording of the question or the range of possible responses. Objective measures from chemical analysis or the use of standard measures such as the CCC, EPDS or IPSM may partially alleviate these problems and increase comparability, but inevitably a large proportion of the exposome data is likely to be more ad hoc in nature.

The results described in this paper suggest possible recommendations for future exposome-wide analyses. Greater consistency in the variables appearing in the final models was achieved with imputed data and subset regression. The use of standard forwards or backwards stepwise techniques tended to identify suboptimal models. If orthogonal factor analysis is appropriate, the derived factor scores will exhibit consistency across stepwise methods and selection criteria.

While in this study we have concentrated on the combined CCC score, it is probable that further information can be obtained by considering the individual subscales. In a study of autistic spectrum disorder (ASD), the *Coherence* subscale was one of the strongest traits characterising 80 ASD cases [74]. Results from a factor analysis of traits related to ASD also showed that the 7 CCC subscales contributed to three of the seven identified factors. These factors had important and independent effects in describing the ASD cases compared to a population-based sample of non-ASD children.

## Strengths and limitations

This study is hypothesis free. It is built on the assumption that the development of speech and language is associated with environmental as well as genetic effects. Consequently, we have started analysing the very detailed information collected in the ALSPAC study to identify the environmental factors most associated with the child’s communication ability. In this paper we concentrate on the features identifiable in pregnancy so that the biases inherent in information collected after the child has been identified as having a developmental problem can be avoided. The limitations of this approach are that it is hypothesis generating, and needs other studies to confirm our findings.

In addition, it is important to note that our study cannot unambiguously interpret associations as indicating a direct cause from a risk factor to child communication skills. For instance, pleiotropic genetic effects may produce communication impairments but also some other phenotype such as separation anxiety. Similar effects would tend to be produced in the child creating an artificial (or false positive) association between maternal anxiety and child communication impairments as a result of inheritance or, in other words, the correlation between the mother and the child’s genotype. To rule out such an explanation we would need either an adoption study that dissociated shared postnatal environment from shared genes in a family, or direct measures of relevant genotypes as well as shared environments. Nevertheless, against this explanation, we would expect to observe some communication difficulties in the parents as well. We found little association between parental history of speech therapy and the child’s CCC outcome (p = 0.68 and 0.057 for the mother and father respectively). But in general, the possibility exists that the identified factors may confound the true causal associations related to other unobserved factors. The exposome-wide approach and the richness of the ALSPAC database minimises this risk. Furthermore, for most of the environmental factors identified here, other studies have reported similar associations making a causal effect more plausible. However, these studies tended to examine the post-natal period possibly indicating that stronger associations will be observed with equivalent measures after birth.

Other disadvantages with this study are as follows. First, we have no information concerning the communication skills of the parents themselves; a factor that has been shown to be related to communication impairments in a number of studies [75]. However, as noted above, using data on speech therapy for the parents in their own childhoods showed little association with the CCC score of their children. Second, we have only used the 621 environmental factors that were statistically significant at the FDR level, and thus may have missed important variables at a less stringent level of significance. However, sensitivity analyses suggested that the final models derived from a wider selection of variables significant at the 0.1% level still included variables within the 621 identified for the main analyses. Third, with a large number of exposures being considered, it is possible that some may not be measured accurately enough to produce reliable results. This can arise as a combination of the observed effect under-estimating the true effect and the increased error in prediction. A source of this error may be parental report. It is conceivable that parents who have poor communication skills themselves may have been unable to complete the questionnaires accurately. However, the correlation between maternal CCC report and an indicator of language impairments [76], non-word repetition assessed by trained personnel at 8y, was highly significant. In addition, the vast majority of the data was gathered prospectively reducing any recall bias.

## Conclusions

We have identified a number of variables in pregnancy associated with the risk of communication difficulties in children. While we have suggested post-hoc plausible mechanisms and used a stringent level of significance, it is still possible that some of these associations may have occurred by chance or that the identified variables are only markers for the true causal factors. But if replicated in other studies, together these results may suggest possible interventions in pregnancy to improve the child’s development of communication skills. Meanwhile, further analyses will be undertaken to explore the mechanisms by which the predictors identified in our final model influence language skills in the child over time.

## Supporting Information

S1 Methods Description of the exposome version 3.01. (DOCX)

S2 Methods Data related to the partner. (DOC)

S3 Methods Model consistency. (DOC)

S1 Results Missing value imputation. (DOC)

S2 Results Data characteristics and Model selection. (DOC)

S3 Results Factor analysis. (DOC)

S1 Table Final model of 19 variables showing regression coefficients by observed data only and with imputed data (single and multiple imputations). (DOCX)

S2 Table Comparison of domain specific models of CCC score at differing levels of significance using different stepwise techniques. (DOCX)

S3 Table Comparison of final models of CCC score at differing levels of significance using different stepwise techniques. (DOCX)

S4 Table Correlations between variables in the final model (N = 7613). (DOC)

S5 Table Stepwise regression analyses of CCC score on 52 factors (N = 7613). (DOCX)

S6 Table Decision tree analysis (CHAID) of CCC score. (DOC)
