# Supplementary material for: Preconception and Prenatal Environmental Factors Associated with Communication Impairments in 9 Year Old Children Using an Exposome-Wide Approach
Source: PLoS One. 2015 Mar 4;10(3):e0118701. doi: 10.1371/journal.pone.0118701 (PMC4349447; doi:10.1371/journal.pone.0118701)
Supplement: S1 Methods — (DOCX) [file pone.0118701.s001.docx]

Appendix: Description of the exposome Version 3.01

This is a brief description of over 21,000 measures currently included in the exposome from the parents’ childhoods to age 6y of the ALSPAC study child. While categorised as phenotypes and exposures, it is to be noted that phenotypes of one individual can be an exposure to another e.g. maternal depression is a maternal phenotype but an exposure to the child. It is also to be recognised that there is a time dimension to these measures e.g. relationship of parents with the child would be restricted to the grandparent-parent interaction in the preconception and prenatal periods. Sometimes measures related to multiple sub-domains e.g. maternal education. In such situations, the most appropriate was chosen although this was to some extent an arbitrary decision.

**Phenotypes**

1. Personal characteristics
   1. Demographic (dob, ethnicity, handedness)
   2. Anthropometry
   3. Personality (temperament, self-esteem)
   4. Attitudes and beliefs
2. Health
   1. Indicators of general health
      1. Subjective assessment of health
      2. Use of healthcare services
      3. Number of medications
   2. Psychiatric disorders
   3. Infections
   4. Atopic and allergic
      1. Eczema
      2. Hay fever
      3. Ashtma
   5. Gastro-intestinal problems
      1. Constipation
      2. Diarrhoea
      3. Blood in stools
      4. Nausea
      5. Vomiting
   6. Hearing
      1. Hearing loss
      2. Grommets
   7. Incontinence
3. Development
   1. Bowel habits
   2. Hearing
   3. Vision
   4. Behaviour
   5. Motor
   6. Language
   7. Social
   8. Puberty
4. Education
   1. SATs
   2. GCSEs

**Exposures**

1. Socio-economic environment
   1. Occupation
   2. Income
   3. Educational status
   4. Housing tenure
   5. Other
2. Lifestyle
   1. Diet
   2. Smoking
   3. Hard drugs
   4. Activity
3. Home environment
   1. Physical
      1. Amenities
      2. Household composition
      3. Ventilation
      4. Problems
      5. Toys and books
   2. Residential mobility
   3. Relationship between parents
   4. Relationship of parents with child
   5. Pets
   6. Family adversity
4. Other social environments
   1. School
   2. Work
   3. Neighbourhood
   4. Support from family and friends
   5. Religious community
5. Life events
   1. Hospital admissions
   2. Surgery
   3. Accidents & injuries
   4. History of pregnancies
   5. Abuse
   6. Other
6. Chemical and other exposures
   1. Pollutants
      1. Chemicals and fumes
      2. EM radiation
      3. ETS
      4. Biosamples – toxic metals
   2. Diet related
      1. Nutrients
      2. Caffeine
      3. Alcohol
      4. Biosamples – e.g. iodine, fatty acids, lipoproteins
   3. Tar and nicotine
   4. Medical procedures (X-rays, ultrasound etc)
   5. Specific medications
