# Supplementary material for: Preconception and Prenatal Environmental Factors Associated with Communication Impairments in 9 Year Old Children Using an Exposome-Wide Approach
Source: PLoS One. 2015 Mar 4;10(3):e0118701. doi: 10.1371/journal.pone.0118701 (PMC4349447; doi:10.1371/journal.pone.0118701)
Supplement: S2 Methods — (DOC) [file pone.0118701.s002.doc]

**Methods S2**

*Data related to the partner*

Within the set of 621 variables there were 85 variables reporting characteristics of the partner. Of these, 42 were reported by the mother and 43 by the partner himself. In addition, a *Current partner* variable was reported by the mother. It is this group of 43 variables which require special treatment in the model building using the *Current partner* variable as described in the Statistical Analyses section. A further 37 variables were reported by the partner of which 21 related to the mother, 5 related to the neighbourhood or the home and 11 related to the social support. These variables required no special treatment. While it is possible that the partners responded to social support questions solely from a personal perspective (and hence could be assume to be zero when the partner was absent), we took the view that responses related to the family.

There was some disagreement between *Current partner* and the availability of partner data. Of the 11,104 mothers with corresponding partner data, 96% reported currently having a partner but 63 mothers did not. In addition, 37 mothers failed to answer this question and 324 mothers failed to return the associated questionnaire. These 424 mothers had *Current partner* changed to *yes*. Of the 2,867 mothers without partner data, 74% were *yes*, 9.9% *no* with the remainder being missing. This latter group of 471 mothers had *Current partner* imputed.

There was also some disagreement between maternal reported partner variables and *Current partner*. After the above changes, 266 out of the 285 mothers without a current partner reported characteristics for the partner. This was interpreted as describing the absent father. Of the 471 mothers with *Current partner* missing, 69% reported partner characteristics. While this most likely implied a current partner, *Current partner* was left as missing due to the possibility that these data related to an absent father. For the missing value imputation, these data were used to impute other characteristics of the partner whether present or absent.

In modelling the CCC outcome, the characteristics of an ex-partner were assumed to have no impact on the pre-natal environment other than via a global effect of no current partner. This may reflect an over-simplification of a range of behaviours by the ex-partner from no contact at all to a supportive and caring friendship. However, any positive aspects are likely to be less than those associated with a current partner.

Three questions related to the neighbourhood, *Clean*, *Attractive* and *Friendly*, had two versions in the partner questionnaires with two responses (yes/no) or three responses (usually/sometimes/never). These data were combined with values assigned to the two responses so that the averages from both forms were identical. In practice, due to frequencies of the responses, this approach tended to position *no* about halfway between *never* and *sometimes* with *yes* tending to occur closer to *usually* than *sometimes* in the ratio of about 1:2. Analyses with the transformed data (N~5430) showed larger regression coefficients in predicting the CCC score than without (N=4671) but the increase was relatively marginal (<8%). This combined with smaller SEs due to the increased sample size, improved the statistical significance of results.
