# Supplementary material for: Preconception and Prenatal Environmental Factors Associated with Communication Impairments in 9 Year Old Children Using an Exposome-Wide Approach
Source: PLoS One. 2015 Mar 4;10(3):e0118701. doi: 10.1371/journal.pone.0118701 (PMC4349447; doi:10.1371/journal.pone.0118701)
Supplement: S3 Methods — (DOC) [file pone.0118701.s003.doc]

**Methods S3**

*Model consistency*

Stepwise regression is a common tool for selecting a subset of variables which best predicts an outcome of interest. While it is well known that this technique tends to ‘over-fit’ the data, it is less clear how robust the model selection is in terms of consistency within a particular data set. So whereas over-fitting is concerned primarily with the exaggerated significance of selected variables leading to a lack of replication in other studies, we are concerned here with whether different criteria applied to one data set will lead to a set of models which are nevertheless consistent in some pre-defined fashion.

One measure of consistency is whether different stepwise methods produce the same final model. Forwards, backwards and subset regressions will be compared. While the traditional forwards and backwards techniques are applied to all eligible variables, in practice they only consider a fraction of all possible models. As a consequence, it has been reported that they often fail to identify the best model. Subset regression based upon random subsets of the eligible variables allows more of the possible models to be compared. However, it is rarely possible to consider all possible models since this can represent trillions of combinations. Each subset is likely to include some redundant variables and hence backwards elimination is applied. The subset size was chosen as the maximum number of variables from forwards and backwards regression plus three.

A second measure is whether the same variables appear when the inclusion/removal criteria are weakened. It is expected that this change would increase the number of variables selected but it would only be consistent if the extra variables represented additional variables. These models are compared using the FDR, 0.001 and 0.01 criteria.
