# Supplementary material for: Preconception and Prenatal Environmental Factors Associated with Communication Impairments in 9 Year Old Children Using an Exposome-Wide Approach
Source: PLoS One. 2015 Mar 4;10(3):e0118701. doi: 10.1371/journal.pone.0118701 (PMC4349447; doi:10.1371/journal.pone.0118701)
Supplement: S1 Results — (DOC) [file pone.0118701.s004.doc]

**Results S1**

*Missing value imputation*

Missing data accounted for 16% of the total number of data points varying between 0% (*Maternal age*) to 28% (*Maternal social class*) for 541 maternal variables and from 20% (*In trouble with law*) to 49% (*Preferred method of feeding*) for the 80 partner reported variables.

Some missing data arose because certain questions were not applicable to the mother or the partner. For instance, for 2.1% of mothers without a partner, data would not be available for partner characteristics on 85 variables of which 42 were reported by the mother. While arguably it was not necessary to impute values for ex-partners, for ease of computation, data were computed as if all mothers had a current partner. This approach utilised all available data on the last partner even if not current (see Methods S2) which may improve the imputation of the current partner status (by comparison of the data for current/ex- partners) and improve the reliability of imputation equations by increasing the amount of observed data. In contrast, for nine variables which measured exposures, type of activities and travelling in relation to work, missing values were set to minimum values when the mother was not working. Similarly the age of starting to smoke was missing for non-smokers. For these variables, a new category was created such that the possible responses were <14 years, 14 or 15, 16 to 19, 20+ and non-smoker.

Within the selected variables there were a number of score variables derived from a number of other variables. To overcome the linear dependency, the combined score was defined as a passive variable in the imputation process and derive post imputation. This strategy sometimes required the inclusion of non-selected items in the imputation process.

While it was considered preferable to utilise as much data as possible in this estimation procedure, it was recognised that different correlations between the variables may exist for those families which participated at 9 years to provide the CCC data and other families. To investigate this further, the imputation was repeated for just the 7613 children. Overall 7% of the data points were missing ranging from 0% to 16.7% (*Partner tar intake*) for maternal variables and from 20.3% to 49.2% for partner variables. The two imputations produced highly consistent results with all correlations >0.61 and with 89% of variables having r>0.9 (max imputed N = 3744).

Single imputations were derived by performing as many cycles as necessary until the maximum difference between imputations in adjacent cycles was <0.05 for any variable. Predicted values from the imputation equations at each iteration replaced previous imputed values. These estimates were constrained to lie within the range of observed values. Multiple imputations were derived using 10 cycles each involving 621 imputation equations. Error components were applied to the predicted values from the final equations reflecting the error in estimating the equation coefficients and the error in predicting the outcome. Twenty multiple imputations were calculated using randomly generated initial imputed values. Stata’s **mi impute** command was used to generate multiple imputations. Although it is common practice to include the outcome for the main analysis in the imputation process (the CCC score in this study), we took a more cautious approach and excluded this variable. As a consequence, imputed values will not include any variability solely associated with the CCC score. However, this approach guaranteed that any subsequent association with the CCC score was not an artefact of the imputations being based upon this variable.

The impact of imputation on the final model is shown in Table S1. In general, the effect sizes were similar although *Feel good score*, *Lowest level of accommodation* and *Bending a lot pre-pregnancy* decreased by 53-60% with complete data compared to the imputed data model. It is estimated that imputation increased the error variance by 7.7%.
