# Supplementary material for: Preconception and Prenatal Environmental Factors Associated with Communication Impairments in 9 Year Old Children Using an Exposome-Wide Approach
Source: PLoS One. 2015 Mar 4;10(3):e0118701. doi: 10.1371/journal.pone.0118701 (PMC4349447; doi:10.1371/journal.pone.0118701)
Supplement: S2 Results — (DOC) [file pone.0118701.s005.doc]

**Results S2:** Data characteristics and Model selection

*Domain specific models*

For the 621 variables meeting the FDR criterion in univariable analyses, the variables which were identified as important in domain specific models varied between procedures Forwards and backwards), stepwise criteria and data sets (see Table S2). Subset regression was not considered at this stage.

The backwards method appeared less consistent on stepwise criteria in the imputed data set than the forwards better but more consistent in the observed data set. Greater inconsistencies were observed in the observed data set with 6 domains (compared to 4 with imputed data) selecting different models at the FDR criterion and 13 domains (compared to 4) when criteria were varied. The backwards method tended to produce a better explanation of the CCC score.

While some of the discrepancies might be considered minor, eg the *Eating of pies and pasties* compared to the *Processed dietary factor* in domain 10, others might be more major eg *Any hearing loss* (backwards only) and *Feel uneasy and restless* (forwards) in domain 2. These latter discrepancies tended to occur more frequently in the observed data set.

These differences were attributed to the pattern of missing data. Although the stepwise procedures attempted to use the maximum data available, inevitably the sample size varied at each step with the possibility of effects being attenuated or amplified in the different samples. While it is commonplace to use only complete data in such analyses to minimise these problems, complete data were only available for 44% of cases on average (range 5% for domain VI to 95% for domain III).

*Final models*

Tables S3 summarises the various final models for observed and observed plus imputed data respectively. Again, the use of imputed data increases consistency both in terms of methods and selection criteria. Imputed data also allowed a better explanation of the CCC score even though this outcome was not included in the imputation process. Subset regression results had greater consistency than other methods although the improvement was relatively minor for observed data.

While consistency provides a degree of confidence one can place in a particular stepwise model, it is possible that some of the inconsistencies were due to similar variables substituting for each other in particular models. *Eating pies and pasties* and the *Processed dietary factor* would appear to be an example featuring both in forwards method (both observed and imputed data sets) and subset method (observed data set). In this context, subset regression may have advantages over other stepwise procedures in that it can summarise a range of models rather than just report the ‘best’ model. These alternative models may provide insights into such substitution and provide additional information for model selection.

Factor analysis may provide another solution. While there can be difficulty in interpreting the derived factors, there are clear advantages in model selection. Being orthogonal, the 52 factors exhibited no differences between stepwise methods and no inconsistencies between selection criteria (see Results S3 and Table S5).
