# Supplementary material for: Preconception and Prenatal Environmental Factors Associated with Communication Impairments in 9 Year Old Children Using an Exposome-Wide Approach
Source: PLoS One. 2015 Mar 4;10(3):e0118701. doi: 10.1371/journal.pone.0118701 (PMC4349447; doi:10.1371/journal.pone.0118701)
Supplement: S3 Results — (DOC) [file pone.0118701.s006.doc]

**Results S3**

*Factor analysis*

A factor analysis was performed on the 621 variables meeting the FDR criterion in unadjusted analyses. The data used in this analysis included imputed data since complete data were only available for 1202 cases – 8.6% of 13,971 children with at least one valid observation. Horn’s parallel analysis suggested 82 factors had eigenvalues greater than would be expected by chance (critical value 1.019). These factors explained 85.4% of the total communalities of 433.7 or 81.9% of the total positive eigenvalues. Between 29 and 61 factors were related to the CCC score depending on the level of significance used (see Table S5).

Comparison of factor models with the individual variable model in Table 3 showed many similarities. However the factors tended to represent a more general characteristic of which the individual variable was an example. So for instance, Factor 10 also included variables relating to the partner’s smoking. Similarly, Factor 1 included other measures from the EPDS scale.

But in addition, the factor models suggest additional determinants of CCC scores including smoking by the mother (factor 2), the relationship between the mother and her partner (factor 16), the partner’s subjective health (factors 7 and 27), adverse current life events including major financial problems (factor 26), parental stability for the mother during her childhood (factor 25) and, at α=0.001, aspects of the neighbourhood including more general assessments as well as more specific details relating to fears about crime (factor 55 in addition to factor 74 shown in Table S5).

The 29 factors at the FDR level shared ~10% of the variability in CCC scores with the 19 variables in Table 3. This suggests that some or all of these variables reflect multi-factorial constructs.
