# Supplementary material for: Preconception and Prenatal Environmental Factors Associated with Communication Impairments in 9 Year Old Children Using an Exposome-Wide Approach
Source: PLoS One. 2015 Mar 4;10(3):e0118701. doi: 10.1371/journal.pone.0118701 (PMC4349447; doi:10.1371/journal.pone.0118701)
Supplement: S1 Table — (DOCX) [file pone.0118701.s007.docx]

Table S1: Final model of 19 variables showing regression coefficients by observed data only and with imputed data (single and multiple imputations).

|  | Observed only | Observed & imputed | |
| --- | --- | --- | --- |
| Variable |  | Single | Multiple |
| Maternal education | 0.77 | 0.79 | 0.80 |
| Social network score | 0.60 | 0.70 | 0.72 |
| Feel good score | 0.36 | 0.60 | 0.43 |
| Others will think less of real self | -0.50 | -0.56 | -0.56 |
| Unnecessary self blame | -0.59 | -0.56 | -0.55 |
| Lowest level of accommodation | -0.27 | -0.51 | -0.50 |
| Often unfairly blamed | -0.34 | -0.51 | -0.48 |
| Babies need stimulation to develop | 0.44 | 0.48 | 0.45 |
| Physically abused 0-16y | -0.38 | -0.45 | -0.40 |
| Processed dietary factor | -0.34 | -0.45 | -0.49 |
| Mother argues with neighbours | -0.40 | -0.44 | -0.44 |
| Bending a lot pre-pregnancy | -0.23 | -0.43 | -0.42 |
| Effort would be in vain | -0.64 | -0.47 | -0.45 |
| Mother didn’t want this pregnancy | -0.42 | -0.41 | -0.30 |
| Bottle feeding more convenient | -0.45 | -0.43 | -0.45 |
| Want to know about labour | -0.41 | -0.40 | -0.41 |
| Night coughing in past 2y | -0.34 | -0.40 | -0.41 |
| Ever badly scalded | -0.31 | -0.39 | -0.37 |
| Any hearing loss | -0.45 | -0.38 | -0.37 |
| N | 4672 | 7613 | 7613 |
| R^2^ (%) | 11.29 | 13.34 | 12.92 |
| Error variance | 70.13 | 75.55 | 75.93 |

Variables were standardised to have a variance of 1. All variables in the observed data model were significant at the 1% level except *Lowest level of accommodation* (p=0.027), *Often unfairly blamed* (p=0.011), *Bending a lot pre-pregnancy* (p=0.065) and *Ever badly scalded* (p=0.013). The single imputation results are also given in Table 3. The multiple imputation results relate to the average of 20 imputed data sets. All variables were significant at FDR criterion except *Mother didn’t want this pregnancy* (p=0.0025), *Ever badly scalded* (p=0.00024) and *Any hearing loss* (p=0.00027).
