# Supplementary material for: Preconception and Prenatal Environmental Factors Associated with Communication Impairments in 9 Year Old Children Using an Exposome-Wide Approach
Source: PLoS One. 2015 Mar 4;10(3):e0118701. doi: 10.1371/journal.pone.0118701 (PMC4349447; doi:10.1371/journal.pone.0118701)
Supplement: S2 Table — (DOCX) [file pone.0118701.s008.docx]

Table S2: Comparison of domain specific models of CCC score at differing levels of significance using different stepwise techniques

| Domain | Method | Stepwise criteria | | Best |
| --- | --- | --- | --- | --- |
|  | B=F | B | F | R^2^ |
| **Imputed data** |  |  |  |  |
| 1 | Y | Y | Y | BF |
| 2 | N | N | N | B |
| 3 | Y | Y | Y | BF |
| 4 | Y | Y | Y | BF |
| 5 | N | Y | Y | B |
| 6 | Y | N | Y | BF |
| 7 | Y | Y | Y | BF |
| 8 | N | Y | Y | B |
| 9 | N | N | Y | B |
| 10 | Y | Y | Y | BF |
| **Observed data** |  |  |  |  |
| 1 | Y | N | Y | BF |
| 2 | N | Y | N | B |
| 3 | Y | Y | Y | BF |
| 4 | N | Y | N | B |
| 5 | N | Y | Y | B |
| 6 | N | Y | N | B |
| 7 | Y | N | N | BF |
| 8 | N | Y | N | B |
| 9 | Y | N | N | BF |
| 10 | N | Y | Y | B |

Comparison of forwards and backwards methods is reported for the FDR criterion (p to enter ≤ α, p to remove > α, α = 0.0001572). Y implies the same variables were selected.

Comparisons of stepwise criteria are made for the FDR, 0.001 and 0.1 inclusion/removal criteria reported for forwards and backwards methods separately. Y implies that as the criteria are weakened only additional variables appear in the final model.
