# Supplementary material for: Preconception and Prenatal Environmental Factors Associated with Communication Impairments in 9 Year Old Children Using an Exposome-Wide Approach
Source: PLoS One. 2015 Mar 4;10(3):e0118701. doi: 10.1371/journal.pone.0118701 (PMC4349447; doi:10.1371/journal.pone.0118701)
Supplement: S3 Table — (DOCX) [file pone.0118701.s009.docx]

Table S3: Comparison of final models of CCC score at differing levels of significance using different stepwise techniques

|  | Stepwise methods | | | Across |
| --- | --- | --- | --- | --- |
|  | Forwards | Backwards | Subset | methods ^b^ |
| **Imputed data** |  |  |  |  |
| Number variables ^a^ | 18 | 18 | 19 | 16 |
| Across criteria ^b^ | 13 | 15 | 19 | 11 ^c^ |
| R^2^ (%)^a^ | 13.25 | 13.25 | 13.34 |  |
| N ^a^ | 7613 | 7613 | 7613 |  |
| **Observed data** |  |  |  |  |
| Number variables ^a^ | 13 | 14 | 15 | 6 |
| Across criteria ^b^ | 7 | 8 | 7 | 3 ^c^ |
| R^2^ (%)^a^ | 11.23 | 11.50 | 11.83 |  |
| N ^a^ | 6142 | 6149 | 6031 |  |

^a^ For models using the FDR criterion

^b^ Consistency across the three methods and across the three inclusion/removal criteria (using FDR, 0.001 and 0.01) is reported as the number of variables common to the three models.

^c^ Consistency across all nine models is also reported.
