# Supplementary material for: Preconception and Prenatal Environmental Factors Associated with Communication Impairments in 9 Year Old Children Using an Exposome-Wide Approach
Source: PLoS One. 2015 Mar 4;10(3):e0118701. doi: 10.1371/journal.pone.0118701 (PMC4349447; doi:10.1371/journal.pone.0118701)
Supplement: S4 Table — (DOC) [file pone.0118701.s010.doc]

Table S4: Correlations between variables in the final model (N=7613)

|  | Variable | 1 | 2 | 3 | 4 | 5 | 6 | 7 | 8 | 9 | 10 | 11 | 12 | 13 | 14 | 15 | 16 | 17 | 18 |
| --- | --- | --- | --- | --- | --- | --- | --- | --- | --- | --- | --- | --- | --- | --- | --- | --- | --- | --- | --- |
| 1 | Maternal education | 1.00 |  |  |  |  |  |  |  |  |  |  |  |  |  |  |  |  |  |
| 2 | Social network score | 0.21 | 1.00 |  |  |  |  |  |  |  |  |  |  |  |  |  |  |  |  |
| 3 | Feel good score | 0.11 | 0.19 | 1.00 |  |  |  |  |  |  |  |  |  |  |  |  |  |  |  |
| 4 | Others will think less of real self | 0.02 | -0.13 | -0.26 | 1.00 |  |  |  |  |  |  |  |  |  |  |  |  |  |  |
| 5 | Unnecessary self blame | -0.07 | -0.13 | -0.24 | 0.25 | 1.00 |  |  |  |  |  |  |  |  |  |  |  |  |  |
| 6 | Lowest level of accommodation | -0.12 | -0.08 | -0.07 | 0.01 | 0.03 | 1.00 |  |  |  |  |  |  |  |  |  |  |  |  |
| 7 | Often unfairly blamed | -0.21 | -0.21 | -0.18 | 0.14 | 0.22 | 0.08 | 1.00 |  |  |  |  |  |  |  |  |  |  |  |
| 8 | Babies need stimulation to develop | 0.18 | 0.07 | 0.08 | 0.00 | -0.03 | -0.07 | -0.07 | 1.00 |  |  |  |  |  |  |  |  |  |  |
| 9 | Physically abused 0-16y | -0.06 | -0.13 | -0.09 | 0.07 | 0.06 | 0.08 | 0.13 | -0.02 | 1.00 |  |  |  |  |  |  |  |  |  |
| 10 | Processed dietary factor | -0.17 | -0.04 | -0.06 | 0.03 | 0.06 | 0.08 | 0.10 | -0.07 | 0.02 | 1.00 |  |  |  |  |  |  |  |  |
| 11 | Mother argues with neighbours | -0.07 | -0.06 | -0.05 | 0.01 | 0.06 | 0.03 | 0.11 | -0.03 | 0.06 | 0.08 | 1.00 |  |  |  |  |  |  |  |
| 12 | Bending a lot pre-pregnancy | -0.05 | -0.02 | -0.03 | 0.04 | 0.09 | 0.01 | 0.07 | 0.02 | 0.04 | 0.04 | 0.04 | 1.00 |  |  |  |  |  |  |
| 13 | Effort would be in vain | -0.26 | -0.22 | -0.18 | 0.12 | 0.17 | 0.12 | 0.34 | -0.12 | 0.09 | 0.09 | 0.10 | 0.04 | 1.00 |  |  |  |  |  |
| 14 | Mother didn’t want this pregnancy | -0.03 | -0.05 | -0.04 | 0.04 | 0.06 | 0.01 | 0.04 | -0.01 | 0.05 | 0.04 | 0.03 | 0.04 | 0.06 | 1.00 |  |  |  |  |
| 15 | Bottle feeding more convenient | -0.29 | -0.11 | -0.10 | 0.01 | 0.09 | 0.06 | 0.13 | -0.10 | 0.01 | 0.10 | 0.03 | 0.01 | 0.16 | 0.04 | 1.00 |  |  |  |
| 16 | Want to know about labour | -0.15 | -0.08 | -0.08 | 0.03 | 0.06 | 0.04 | 0.07 | -0.09 | 0.02 | 0.05 | 0.02 | 0.01 | 0.12 | 0.02 | 0.14 | 1.00 |  |  |
| 17 | Night coughing in past 2y | -0.10 | -0.05 | -0.11 | 0.05 | 0.12 | 0.07 | 0.13 | -0.06 | 0.07 | 0.10 | 0.09 | 0.06 | 0.14 | 0.03 | 0.05 | 0.05 | 1.00 |  |
| 18 | Ever badly scalded | -0.03 | -0.03 | -0.03 | 0.02 | 0.04 | 0.00 | 0.07 | -0.01 | 0.05 | 0.00 | 0.02 | 0.03 | 0.04 | 0.05 | 0.01 | 0.01 | 0.02 | 1.00 |
| 19 | Any hearing loss | 0.01 | -0.05 | -0.09 | 0.07 | 0.07 | 0.00 | 0.06 | -0.01 | 0.04 | 0.03 | 0.00 | 0.02 | 0.02 | 0.01 | 0.01 | 0.01 | 0.07 | 0.02 |
