# Supplementary material for: Preconception and Prenatal Environmental Factors Associated with Communication Impairments in 9 Year Old Children Using an Exposome-Wide Approach
Source: PLoS One. 2015 Mar 4;10(3):e0118701. doi: 10.1371/journal.pone.0118701 (PMC4349447; doi:10.1371/journal.pone.0118701)
Supplement: S5 Table — (DOCX) [file pone.0118701.s011.docx]

Table S5: Stepwise regression analyses of CCC score on 52 factors (N=7613)

| α (R^2^) | Factor | Main variable from Table 2 | B [95% CI] |
| --- | --- | --- | --- |
| FDR | 1 | Unnecessary self blame | -1.05 [-1.25,-0.85] |
| (11.0%) | 2 | Smoked cigarettes 3^rd^ trimester | -0.75 [-0.98,-0.52] |
|  | 3 | CCEI Depression 18w | -0.76 [-0.96,-0.55] |
|  | 6 | Allowed to do as liked 0-16y ^a^ | -0.54 [-0.74,-0.33] |
|  | 7 | Partner change in Feel Good score | 0.56 [0.32,0.80] |
|  | 9 | Total sensitivity ^b^ | 0.55 [0.34,0.75] |
|  | 10 | In smoky room during weekday | -0.56 [-0.78,-0.35] |
|  | 11 | Maternal education ^c^ | 1.02 [0.83,1.21] |
|  | 13 | Photocopier or fax use ^d^ | -0.70 [-0.90,-0.51] |
|  | 15 | Partner has university degree | 0.56 [0.37,0.75] |
|  | 16 | Mother listens to partner’s feelings | 0.41 [0.22,0.60] |
|  | 17 | Bottle feeding more convenient | -0.71 [-0.92,-0.50] |
|  | 21 | Social network score | 0.94 [0.74,1.15] |
|  | 25 | Grandmother in household 0-5y | 0.50 [0.26,0.73] |
|  | 26 | Difficulty in affording things for baby | -0.45 [-0.66,-0.25] |
|  | 27 | Partner looking attractive | 0.60 [0.41,0.80] |
|  | 28 | Feel good score | 0.63 [0.42,0.84] |
|  | 32 | Any hearing loss | -0.45 [-0.65,-0.26] |
|  | 33 | Allowed to do as liked 0-16y ^a^ | 0.44 [0.25,0.64] |
|  | 34 | Processed factor score | -0.66 [-0.86,-0.46] |
|  | 36 | Night coughing in past 2y | -0.60 [-0.80,-0.40] |
|  | 38 | Loss of appetite | -0.52 [-0.73,-0.32] |
|  | 41 | Effort would be in vain ^e^ | -0.69 [-0.90,-0.49] |
|  | 45 | Total milk intake | -0.47 [-0.69,-0.25] |
|  | 46 | Partner Locus of control score | -0.41 [-0.62,-0.21] |
|  | 52 | Cola intake | -0.51 [-0.76,-0.27] |
|  | 70 | EPDS depression 18w | -0.46 [-0.67,-0.25] |
|  | 75 | Has O level | 0.42 [0.21,0.62] |
|  | 81 | Life events score | -0.61 [-0.84,-0.38] |
| 0.001 | 18 | Physically abused 0-16y | -0.38 [-0.58,-0.19] |
| (12.6%) | 74 | Mother argues with neighbours | -0.45 [-0.69,-0.21] |
|  | 78 | Ever badly scalded | -0.34 [-0.54,-0.14] |
|  |  | (8 other factors not shown) |  |
| 0.01 | 60 | Mother didn't want this pregnancy | -0.29 [-0.52,-0.07] |
| (13.9%) |  | (11 other factors not shown) |  |
| 0.05 | 5 | Want to know basics about labour | -0.39 [-0.67,-0.12] |
| (14.4%) | 37 | Lowest level of accommodation | -0.28 [-0.50,-0.06] |
|  |  | (7 other factors not shown) |  |

^a^ Factors 6 and 33 reflected the care and overprotective subscales of the Parental Bonding instrument. Although this variable is typically associated with the overprotective scale, in these data, this variable loaded on both scales.

^b^ *Others will think less of real self* also loaded on this factor.

^c^ *Babies need stimulation to develop* also loaded on this factor.

^d^ *Bending a lot pre-pregnancy* also loaded on this factor.

^e^ *Often unfairly blamed* also loaded on this factor.

Factors are grouped into 4 models using four levels of significance (FDR, 0.1%, 1%, 5%). All factors at one level of significance were included in the final model at a less stringent level of significance. Due to the near orthogonal nature of factors, effects only varied marginally with the inclusion of other factors. The FDR criterion was 0.0001572. Forwards and backwards stepwise regressions produced the same final model.
