# Supplementary material for: Preconception and Prenatal Environmental Factors Associated with Communication Impairments in 9 Year Old Children Using an Exposome-Wide Approach
Source: PLoS One. 2015 Mar 4;10(3):e0118701. doi: 10.1371/journal.pone.0118701 (PMC4349447; doi:10.1371/journal.pone.0118701)
Supplement: S6 Table — (DOC) [file pone.0118701.s012.doc]

Table S6: Decision tree analysis (CHAID) of CCC score

| From | Variable | Node characteristics | | | | | R^2^ |
| --- | --- | --- | --- | --- | --- | --- | --- |
| Node |  | ID | Categories | N | Mean | SD | (%) |
|  | Family adversity index | 1 | 0 | 3547 | 219.3 | 8.19 | 4.09 |
|  |  | 2 | 1 | 2018 | 217.8 | 8.81 |  |
|  |  | 3 | 2 – 3 | 1044 | 216.3 | 9.87 |  |
|  |  | 4 | 4+ | 1004 | 213.7 | 11.77 |  |
| 1 | Maternal education | 5 | None/Vocational | 529 | 216.8 | 8.47 | 0.80 |
|  | (Highest qualification) | 6 | O level | 1287 | 219.1 | 7.86 |  |
|  |  | 7 | A level | 987 | 219.8 | 8.33 |  |
|  |  | 8 | Degree | 744 | 220.7 | 7.94 |  |
| 2 | Effort would be in vain | 9 | Yes | 253 | 214.0 | 9.73 | 0.62 |
|  |  | 10 | No | 1765 | 218.3 | 8.54 |  |
| 3 | Often absent from school | 11 | Yes | 288 | 213.0 | 12.11 | 0.64 |
|  | 11-16y | 12 | No | 756 | 217.5 | 8.55 |  |
| 4 | Effort would be in vain | 13 | Yes | 297 | 210.0 | 14.07 | 0.88 |
|  |  | 14 | No | 707 | 215.3 | 10.27 |  |
| 5 | Major group of last job | 15 | Non-manual | 192 | 219.3 | 6.16 | 0.30 |
|  |  | 16 | Manual | 337 | 215.3 | 9.24 |  |
| 6 | Never sure if others are | 17 | Like me | 377 | 217.1 | 9.04 | 0.33 |
|  | pleased | 18 | Unlike me | 910 | 219.9 | 7.16 |  |
| 7 | Dissatisfied about body | 19 | No/Occasionally | 923 | 220.1 | 7.65 | 0.23 |
|  | shape | 20 | Yes, mostly | 64 | 215.2 | 14.34 |  |
| 8 | Partner – others will not | 21 | Like me | 65 | 217.5 | 10.13 | 0.17 |
|  | Like true self | 22 | Quite unlike me | 125 | 222.7 | 5.12 |  |
|  |  | 23 | Very unlike me | 554 | 220.7 | 8.05 |  |
| 9 | Housing tenure | 24 | Mortgaged/owned | 76 | 211.1 | 12.33 | 0.14 |
|  |  | 25 | Rented/other | 177 | 215.3 | 8.10 |  |
| 10 | Processed dietary factor | 26 | ≤ 1.065 | 1586 | 218.7 | 8.09 | 0.36 |
|  |  | 27 | > 1.065 | 179 | 214.8 | 11.24 |  |
| 11 | Number of people to | 28 | 0 or 1 | 51 | 206.9 | 14.98 | 0.35 |
|  | Confide in | 29 | 2+ | 237 | 214.3 | 11.00 |  |
| 12 | Feel uneasy and restless | 30 | Often/Very often | 132 | 214.1 | 10.79 | 0.29 |
|  |  | 31 | Never/Not often | 624 | 218.3 | 7.81 |  |
| 13 | Usually use car | 32 | Yes | 132 | 213.9 | 9.73 | 0.54 |
|  |  | 33 | No | 165 | 206.9 | 16.12 |  |
| 14 | Partner locus of control | 34 | 0 – 5 | 447 | 216.8 | 8.36 | 0.43 |
|  |  | 35 | 6 – 11 | 260 | 212.7 | 12.50 |  |
|  | Total |  |  | 7613 | 217.7 | 9.33 | 10.15 |

Nodes 15 to 35 are terminal nodes.

All variables are initially split into 10 categories and then subsequently adjacent categories are combined based upon comparisons of the mean CCC scores. SDs reflect the variability of the CCC score within each node or category.

R^2^s reflect the additional contribution of each variable to the explanation of the CCC score. The total explanation was 10.2% with 21 dfs.
